# Comparative Study of the Antioxidant and Anti-Inflammatory Effects of Leaf Extracts from Four Different *Morus alba* Genotypes in High Fat Diet-Induced Obesity in Mice

**DOI:** 10.3390/antiox9080733

**Published:** 2020-08-11

**Authors:** Francisco Javier Leyva-Jiménez, Antonio Jesús Ruiz-Malagón, José Alberto Molina-Tijeras, Patricia Diez-Echave, Teresa Vezza, Laura Hidalgo-García, Jesús Lozano-Sánchez, David Arráez-Román, José Luis Cenis, Antonio Abel Lozano-Pérez, Alba Rodríguez-Nogales, Antonio Segura-Carretero, Julio Gálvez

**Affiliations:** 1 Research and Development Functional Food Centre, Health Science Technological Park, Avenida del Conocimiento 37, E-18016 Granada, Spain; jleyva@cidaf.es (F.J.L.-J.); jesusls@ugr.es (J.L.-S.); darraez@ugr.es (D.A.-R.); ansegura@ugr.es (A.S.-C.); 2 CIBER-EHD, Department of Pharmacology, Center for Biomedical Research (CIBM), University of Granada, 18071 Granada, Spain; a.jesus.ruiz14@gmail.com (A.J.R.-M.); jalbertomolinatijeras@gmail.com (J.A.M.-T.); pdiezechave@gmail.com (P.D.-E.); teresavezza@hotmail.it (T.V.); lhidgar@gmail.com (L.H.-G.); jgalvez@ugr.es (J.G.); 3 Instituto de Investigación Biosanitaria de Granada (Ibs. GRANADA), 18071 Granada, Spain; 4 Department of Nutrition and Food Science, Center for Biomedical Research (CIBM), University of Granada, 18071 Granada, Spain; 5 Department of Analytical Chemistry, University of Granada, 18071 Granada, Spain; 6 Departamento de Biotecnología, Genómica y Mejora Vegetal, Instituto Murciano de Investigación y Desarrollo Agrario y Alimentario, 30150 La Alberca (Murcia), Spain; josel.cenis@carm.es; 7 Servicio de Digestivo, Hospital Universitario Virgen de las Nieves, 18012 Granada, Spain

**Keywords:** *Morus alba* leaves, high fat diet, obesity, chemical characterization, anti-inflammatory, metabolic disorders

## Abstract

Increased levels of reactive oxygen species (ROS) and a low-grade chronic inflammation in multiple organs have been demonstrated in obesity. *Morus alba* leaves extracts (MAEs) have been used in traditional medicine as anti-inflammatory agents. In this work, the bioactive compounds of different genotypes of *M. alba L.* (*Filipina*, *Valenciana Temprana*, *Kokuso*, and *Italia*) were analyzed not only by reverse phase high performance liquid chromatography–electrospray ionization-time of flight-mass spectrometry (RP-HPLC-ESI-TOF-MS) and hydrophilic interaction chromatography–electrospray ionization-time of flight-mass spectrometry (HILIC-ESI-TOF-MS), but also screened for in vitro and in vivo antioxidant activity by means of DPPH• radical scavenging assay and *Caenorhabditis elegans* model. These MAEs were administered daily in a model of diet-induced obesity in mice. *Filipina* and *Italia* genotypes significantly reduced weight gain, the glycemic levels in high fat diet, as well as, levels of LDL-cholesterol and triglycerides. *Filipina* and *Italia* MAEs also reduced the expression of proinflammatory mediators such as *Tnf-α*, *Il-1β*, *Il-6* and increased the levels of adiponectin and AMPK, which exert anti-inflammatory effects. Moreover, *Italia* genotype ameliorated the intestinal barrier function. In conclusion, *Filipina* and *Italia* methanolic extracts show the highest antioxidant and anti-inflammatory effect, due to the presence of compounds such as protocatechuic acid or quercetin-3-glucoside, and they could be developed as a complementary treatment for obesity and metabolic disorders.

## 1. Introduction

According to recent epidemiologic reports, the prevalence of obesity is reaffirming an alarming rate all over the world. Results show an increasing prevalence in both women and men, and also in children, estimating that 107 million children and 603 million adults were obese in 2015, considering obesity as an epidemic [1]. Obesity is defined as excessive body fat accumulation caused by a positive energy balance, that could lead a cluster of conditions such as high blood pressure, insulin resistance or dyslipidemia, being closely related to metabolic syndrome, which causes organic disorders, increasing the risk to suffer diabetes or liver and heart diseases [2]. In obese individuals, the presence of low-grade chronic inflammation in multiple organs has been demonstrated and its origin has been related to changes in cells of metabolic tissues in response to excess nutrients and energy [3]. These disorders can be ameliorated with an improved lifestyle, including exercise and healthy food habits, and may include pharmacological approaches. When considering the latter, the irregular weight loss obtained and the costs of pharmacotherapy, including the risks of serious adverse effects, make this strategy an unsuccessful alternative. Therefore, in the last decades, efforts have been focused on the discovery of safer and more effective treatments, like those involving bioactive compounds from natural sources capable of ameliorating obesity [4].

In this scenario, mulberry, *Morus alba L.*, is a deciduous tree native from the foothills of the Himalayas in China and disseminated first to Korea and Japan and after to India, Persia, Europe, and South and North America [5] due to its great genetic variability and adaptability to different environmental conditions [6]. Mulberry leaves have been traditionally used as infusions in Traditional Chinese Medicine to alleviate fever, strengthen joints, or treat constipation and have gained growing interest worldwide due to their bioactive components [7,8]. Mulberry plantations spread along the “Silk Road”, as the use of silkworms to produce silk for textile applications reached southern European countries such as Italy, France, Spain, or Portugal. The importance of *M. alba L.* arises from the fact that their leaves are the only food source of the silkworm (*Bombyx mori L.*) As a consequence, they are an essential part of sericulture activity, what is still very important in Asian countries, such as China, India, and Pakistan, among others [6]. *M. alba* leaves present considerable nutritional value and high-quality proteins. They are often used in India as a dietary supplement by mixing with flour and as a ruminant food [9].

Recent studies have reported several bioactive properties of *M. alba* such as antibacterial, antioxidant, anti-obesogenic, or hypoglycemic activities [10,11,12,13]. The healthy properties of mulberry have been related to the content of phytochemical in its leaves and fruits, such as flavonoids or phenolic acids [14].

The composition of mulberry leaves is strongly related to the harvest season, geographic location, and the studied variety of *Morus* [15]. Additionally, the extraction method applied in order to obtain a bioactive extract can also be crucial for the final chemical composition. In this sense, plant extracts with bioactive compounds have been traditionally obtained through conventional extractions such as maceration or infusion [16], but innovative techniques such as pressurized liquid extraction (PLE) are claimed as better in extraction yield and in terms of variety of obtained compounds [17]. Actually, the chemical characterization of the phytochemicals in *M. alba* leaf extract can be a complex task since it has a huge variety of structures including phenolic compounds and sugars. Generally, C18 reversed-phase high-performance-liquid chromatography (RP-HPLC) has been applied to characterize the phenolic fraction [5] coupled to different detectors in order to ensure an exhaustive characterization such as diode-array detection (DAD) and mass spectrometry (MS).

In this study, the extracts containing bioactive compounds from four genotypes of *M. alba* (*Italia*, *Kokuso*, *Filipina*, and *Valenciana Temprana*) were retrieved from leaves using PLE and characterized applying RP-HPLC coupled to time-of-flight mass spectrometry, in order to determine the chemical composition of all the extracts. The obtained extracts were screened for in vivo antioxidant activity in a *C. elegans* model and also tested in a diet-induced obesity mice model in order to compare the modulation of obesity-related biochemical markers regulated by all genotypes. Furthermore, the comprehensive characterization enabled to establish a relation between chemical composition and beneficial effects against experimentally-induced obesity.

## 2. Materials and Methods

### 2.1. Reagents

All reagents used in this research were analytical or HPLC grade and were used without further purification. As far as the extraction procedure is concerned, water was purified by a Milli-Q system from Millipore (Bedford, MA, USA) and ethanol was purchased from VWR chemicals (Radnor, PA, USA). The analytical procedure was performed using: (a) LC-MS grade acetonitrile and ammonium formate, which were purchased from Fisher chemicals (Waltham, MA, USA); (b) formic acid was purchased from Sigma-Aldrich (Steinheim, Germany). The calibration curves were performed using commercial standards (quinic acid, protocatechuic acid, 3,5-methoxy-4-hydroxy cinnamic acid, chlorogenic acid, rutin, kaempferol-3-rutinoside, quercetin-3- glucoside, kaempferol-3-glucoside, and quercetin) purchased either from Fluka, Extrasynthese (GenayCedex, France), or Sigma-Aldrich (Steinheim, Germany). Both 2,2-diphenyl-1-picrylhydrazyl radical (DPPH•) and H_2_O_2_ were purchased from Merck (Merck KGaA, Darmstadt, Germany).

### 2.2. Plant Materials and Extraction Procedure of Bioactive Compounds from M. alba

Four *M. alba* genotypes, as representative examples of those well acclimated to the Mediterranean environmental conditions, were used in the experiment. All of those are part of the Mulberry Germplasm Bank of the Sericulture Program at the Instituto Murciano de Investigación y Desarrollo Agrario y Alimentario (IMIDA), Murcia (Spain). The references of the genotypes in the collection where: BGMU 050 10009 (*Italia*); BGMU 050 20010 (*Filipina*); BGMU 050 10003 (*Kokuso*); and BGMU 050 10040 (*Valenciana Temprana*) (IMIDA coordinates: Long. 37.9388011, Lat. -1.1333683, Alt. 54 m.). The selected genotypes produce leaves very suitable for feeding silkworms and are tolerant to the dry and warm conditions of the southeast of Spain [18]. The leaves were collected during the first week of May, washed with tap water, and dried over absorbent paper, frozen at −80 °C and lyophilized at −55 °C for 72 h in a Christ Alpha 1-2 LDPlus (Martin Christ, Osterode am Harz, Germany). Dry leaves were grounded by using an ultra-centrifugal mill ZM200 (Restch GmbH, Haan, Germany). After milling, samples were shaded and kept at room temperature until extraction. The extraction of bioactive compounds from mulberry was performed using a pressurized liquid extractor (ASE™ 350 system, Dionex, Sunnyvale, CA, USA) equipped with a solvent controller. The extractions were accomplished using a mixture ethanol/H_2_O 50:50 (*v*:*v*) as solvent, at 200 °C for 20 min in static cycle. The pressure was pre-set at 1500 psi. Briefly, 3.75 g of sample was mixed with 11.25 g of sea sand and loaded onto 33 mL stainless-steel extraction cells. Cellulose filters and two portions of sea sand (5 g) were placed at each end of the cell to avoid clogging of metal frits. After extraction, the obtained extracts were immediately cooled in ice to attain a temperature of 20–25 °C. The extracts were finally filtered through 0.45 µm filters and the supernatants were dried under vacuum in a Savant ™ SpeedVac Concentrator SC250 EXP (Thermo Scientific, Sunnyvale, CA, USA) and frozen at −20 °C until the analysis was performed.

### 2.3. RP-HPLC-ESI-TOF-MS Analysis

Samples were prepared at 2500 mg/L by dissolving the appropriate amount of extract obtained by PLE in the same solvents used in extractions. *M. alba* extracts (MAEs) were analyzed using a RRLC 1200 series (Agilent Technologies, Palo Alto, CA, USA), equipped with a vacuum degasser, autosampler, a binary pump, and a DAD detector. The separation of the phenolic fraction was conducted by a Zorbax Eclipse Plus C18 whose sizes were 150 mm × 4.6 mm id, 1.8 µm (Agilent Technologies, Palo Alto, CA, USA) where the eluent A was water/acetonitrile 90:10 (*v*:*v*) and formic acid added at 0.1%, whereas eluent B was only acetonitrile. The flow was held at 0.5 mL/min during 45 min using the following multistep linear gradient: 0 min, 5% B; 15 min, 65% B; 36 min, 95% B; 40 min, 5% B; and finally, a conditioning cycle of 5 min with the initial conditions for the next analysis. Then, 10 µL of the sample was injected and the separation of the compounds was carried out at 25 °C. 

The HPLC system was coupled to a time-of-flight mass spectrometer (micrOTOF, BrukerDaltonik GmbH, Bremen, Germany) that comprised an electrospray interface (ESI) (model G1607 from Agilent Technologies, Palo Alto, CA, USA) operating in negative ionization mode. To ensure a correct ionization of the analytes, a “T” type splitter was used to reduce the flow towards mass spectrometer. The parameters of the source were optimized and set as follows: capillary voltage of +4 kV; drying gas temperature, 210 °C; drying gas flow, 9 L/min; and nebulizing gas pressure, 2.3 bar. The values of transfer parameters were capillary exit, −120 V; skimmer 1, −40 V; hexapole 1, −23 V; RF hexapole, 80 Vpp; and skimmer 2, −20 V. The detection mass range was from 50 to 1000 *m*/*z*.

External mass spectrometer calibration was performed in quadratic high-precision calibration (HPC) regression mode passing a solution containing sodium acetate clusters (5 mM sodium hydroxide in H_2_O/2-propanol 1/1 (*v*/*v*), with 0.2% of acetic acid). With this method, an exact calibration curve was achieved based on numerous cluster masses, each differing by 82 Da (C_2_H_3_NaO_2_). The calibration solution was injected at the beginning of the run using a Cole Palmer syringe pump (Vernon Hills, Illinois, USA) and all the spectra were calibrated prior to polar compounds characterization. The accurate mass data for the molecular ions were processed through Data Analysis 4.0 software (Bruker Daltonics, Bremen, Germany) which provided a list of possible elemental formulas by using the Generate Molecular Formula TM editor. The latter uses a CHNO algorithm that provides standard functionalities such as minimum/maximum elemental range, and ring-plus double-bond equivalents, as well as a sophisticated comparison of the theoretical with the measured isotope pattern (mSigma value) for increasing the confidence in the suggested molecular formula. According to the literature, the widely accepted accuracy threshold for confirming elemental compositions was established at 5 ppm for most of the compounds [19].

Quantitation of identified analytes was carried out by HPLC-ESI-TOF-MS. For that purpose, nine standards curves were performed to quantify the compounds present in leaves: quinic acid, protocatechuic acid, 3,5-dimethoxy-4-hydroxy cinnamic acid, chlorogenic acid, rutin, kaempferol-3-rutinoside, quercetin-3-glucoside, kaempferol-3-glucoside, and quercetin stock of standards were prepared at 1000 mg/L diluting with methanol to working concentrations.

### 2.4. DPPH•Scavenging Activity 

The radical scavenging activity (RSA) was analyzed following the method described by Yen and Duh [20], with some modifications. Briefly, 50 μL of methanolic extract was added to Eppendorf tubes containing 850 μL of methanol, and then 100 µL of 1 mM DPPH•(in methanol) were added. After 30 min of reaction at 25 °C, protected from light, the scavenging activities of the samples and standards (ascorbic acid, 10–500 µM in methanol) were evaluated by measuring the absorbance at 515 nm, in a Synergy MX UV–Vis spectrometer (BioTek Instruments Inc; Winooski, VT, USA). For each sample concentration tested, the inhibition percentage (I%) of DPPH• in the steady state was determined using the equation:

(1) $$I\%=\left[\frac{\left(Abs_{Blank}-Abs_{Sample}\right)}{Abs_{Blank}}\right]\times 100.$$

where *Abs_Blank_* and *Abs_Sample_* are the absorbance at 515 nm of the blank and the samples respectively. The results were expressed as micromols of ascorbic acid equivalents per gram of dry extract (µmol AAE/g DE).

### 2.5. Oxidative Stress Assays

To validate the potential antioxidant activity of MAEs, *C. elegans* wild type strain N2 (*Caenorhabditis* Genetics Center at the University of Minnesota, Minneapolis, MN, USA) was used as previously reported [21]. For this purpose, *C. elegans* wild type strain N2 was grown in nematode growth medium (NGM: Agar 17.5 g/L, sodium chloride 3.0 g/L, peptone 2.5 g/L, cholesterol 0.005 g/L) on agar plates with (i) a lawn of *E.coli* OP50 (control media); (ii) a lawn of *E.coli* OP50 and Vitamin C (10 µg/mL) (positive control); and (iii) a lawn of *E.coli* OP50 and MAE dissolved in DMSO (100 µg/mL). Worms were incubated at 20 °C for 5 days, and then transferred to a medium with 3 mM of H_2_O_2_. After 5 h of incubation, worm viability was scored. Experiments were performed in triplicate and repeated three times.

### 2.6. Effects of MAEs on High Fat Diet Fed Mice

#### 2.6.1. Animals and Treatments

The study was carried out in accordance with the ‘Guide for the Care and Use of Laboratory Animals’ as promulgated by the National Institute of Health, and the protocols approved by the Ethic Committee of Laboratory Animals of the University of Granada (Spain) (Ref. No. 28/03/2016/030). Male 6-week-old C57BL/6J mice (Janvier, St Berthevin, France) were housed in Makrolon cages (5 mice per cage) with a 12-h light/dark cycle and temperature and humidity-controlled facility (22 ± 1 °C, 55 ± 10% relative humidity). Mice were randomly divided into several groups (*n* = 10): control, obese, and four groups of obese mice treated with four MAEs. Control mice received a normal chow diet (NCD, 13% calories from fat, 20% calories from protein, and 67% calories from carbohydrate) (Global diet 2014; Harlan Laboratories, Barcelona, Spain), whereas obese mice were fed a high fat diet (HFD) in which 60% of their caloric content was derived from fat (Purified diet 230 HF; Scientific Animal Food & Engineering, Augy, France). MAE was dissolved in water (vehicle) and administered daily in a dose of 10 mg/kg by oral gavage. Control and obese groups were daily gavaged with the vehicle. The treatment was followed for 6 weeks, and animal body weight, food, and water intake were monitored regularly every 3 days. These data were used to calculate the energy efficiency, which is the ratio between the final weight gain (g) and the total energy intake (kcal) during the period of the experiment (g/kcal). It was calculated using the formula: weight gain (g)/total energy intake (kcal).

#### 2.6.2. Glucose Tolerance Test

One week before the sacrifice, mice fasted for 8 h underwent a glucose tolerance test. Glucose was administered from a 50% solution in water in a dose of 2 g/kg of body weight by intraperitoneal injection. Blood was collected at 0, 15, 30, 60, and 120 min after treatment from the tail vein and used to measure glucose levels with a handheld glucometer (Contour XT, Ascensia Diabetes Care, S.L., Barcelona, Spain).

#### 2.6.3. Plasma Determinations

Blood samples were collected at the end of the treatment in ice-cold tubes containing heparin. Plasma was obtained after centrifugation at 5000× *g* for 20 min at 4 °C. Plasma glucose, LDL (low-density lipoprotein) cholesterol, HDL (high-density lipoprotein) cholesterol, total cholesterol, and triglycerides concentrations were analyzed by colorimetric methods using Spinreact kits (Spinreact, S.A., Girona, Spain).

#### 2.6.4. Morphological Variables

After mice sacrifice, epididymal and abdominal fat was collected and weighed in order to calculate the fat weight index by dividing the fat weight by the tibia length. All tissue samples were frozen and then stored at −80 °C.

#### 2.6.5. Analysis of Gene Expression by RT-qPCR

RNA was extracted from samples of liver, fat, and colon using NucleoZOL (Macherey-Nagel, Düren, Germany), following the manufacturer’s recommendations. Oligo(dT) primers (Promega, Southampton, UK) were used to carry out reverse transcription. Real time quantitative PCR amplification and detection was performed on optical-grade 48 well plates in EcoTM Real Time PCR System (Illumina, San Diego, CA, USA) with 20 ng of cDNA, the KAPA SYBR ^®^ FAST qPCR Master Mix (KAPA Biosystems, Wilmington, MA, USA), and specific primers whose annealing temperature is showed in Table 1. Glyceraldehyde 3-phosphate dehydrogenase housekeeping gene (*Gapdh*) was used to normalize gene expression. The mRNA relative quantitation was calculated using the ∆∆Ct method.

#### 2.6.6. Histological Studies

Samples of liver and epididymal adipose tissue were fixed in 4% PFA, embedded in paraffin, and 5 µm-thick sections were taken. Then these sections were stained with hematoxylin and eosin. In addition, liver sections were fixed in 30% sucrose, embedded in OCT compound (Tissue-Tek^®^ O.C.T. Compound, Sakura^®^ Finetek, United States), and frozen with isopentane at −40 °C. Then, 8 µm-thick sections were taken and stained with oil red and hematoxylin. An independent pathologist unaware of the experiment evaluated the inflammation and fat accumulation in the histological sections.

### 2.7. Statistics

All results are expressed as the mean ± SEM. Differences between means were assessed for statistical significance using a one-way analysis of variance (ANOVA) and post hoc least significance test. The chi-squared test was used to analyze differences between proportions. All these statistical analyses were carried out with the GraphPad 5.0 software package (GraphPad Software, Inc., La Jolla, CA, USA), the statistical significance was set at *p* ≤ 0.05.

## 3. Results and Discussion

### 3.1. Analytical Characterization of Phytochemicals in MAEs Yielded by PLE Conditions

**Figure 1:**
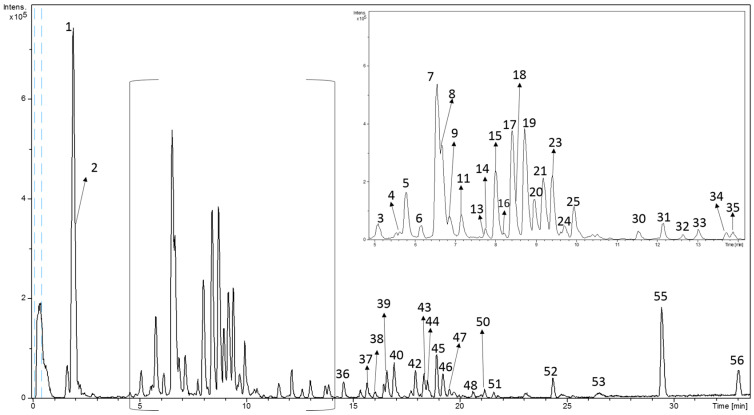
Characterization of extracted compounds of Morus Alba leaves using reverse phase high performance liquid chromatography–electrospray ionization-time of flight-mass spectrometry (RP-HPLC-ESI-TOF-MS).

With the purpose to determine the chemical profile of mulberry extracts and in order to establish compositional differences, a complete RP-HPLC-ESI-TOF-MS analysis was accomplished. Figure 1 includes the MAE chromatograms where the main compounds were numbered in accordance with their elution order. In this sense, a total of 56 compounds were detected in the MAE where the Filipina genotype showed the most complex profile (46 compounds).

Minor number of phytochemicals were determined in *Valenciana Temprana* genotype (38 compounds). The chemical characterization was based on the interpretation of the MS spectra and the data available on the literature. According to their chemical structure, phytochemicals were classified into six groups: organic acids, benzoic acids, cinnamic acids, flavonoids, fatty acids, and others. Unfortunately, the mass data provided by TOF-MS and data suitable on bibliography did not allow the characterization of ten compounds. Thus, Table 2 summarizes the tentative name of compounds accompanied by their number of peaks, retention time (RT), experimental *m*/*z*, deprotonated molecular formula, and also the presence in different MAE.

Regarding the organic acid group, only one compound (peak 2) was detected in all MAE. This compound was characterized as quinic acid according to the displayed molecular formula (C_7_H_12_O_6_) and elution order which were similar to a previous work [22].

Furthermore, the analytical method performed allowed the detection of fourteen phenolic acids. From them, six benzoic acids were successfully characterized. Peak 3 yielded a *m*/*z* at 315.0722 and was tentatively characterized as protocatechuic acid hexoside, being detected in all MAE. Indeed, protocatechuic acid and derivatives have previously been described in the literature [21]. In addition, peak 4 showed a deprotonated molecular formula C_19_H_23_O_13_ and *m*/*z* at 459.1122 allowing its characterization as parishin E. This compound was only detected in *Filipina* MAE. Compound 26 gave a molecular formula C_22_H_30_O_11_. This phytochemical, which was only found in *Italia* MAE, was associated to rengyoside D since it was previously detected in plants from the same class [23]. Besides, two salicylic acid derivatives were also identified. The first eluted derivative was methylsalicyl aldehyde (peak 28). This compound was only found in *Valenciana Temprana* MAE, while the second one (peak 36), which provided a *m*/*z* at 221.1179 and was assigned to hexyl salicylate, was presented in all genotypes. Finally, peak 52, which was detected in all MAEs, was tentatively identified as gingerol [24].

With regard to cinnamic acids, five caffeoylquinic acid derivatives were tentatively characterized in MAE. Four of them (peaks 5, 7, 8 and 9), gave the same molecular formula C_16_H_18_O_9_ and were associated to chlorogenic acid isomers. The elution order enabled the characterization of each isomer by comparison with data compiled from literature [24]. These compounds are found in larger amounts in mulberry extracts [5,22]. Compound 22 with a *m/z* at 515.1186 was characterized as caffeoylquinic acid hexoside [22]. Moreover, peak 31 was characterized as dimethoxy-cinnamic acid since it gave a molecular formula C_11_H_12_O_4_. Its identification was proposed due to different cinnamic acid derivatives previously described in *M. alba* leaves [24]. At last, compound **32** was tentatively attributed to *p*-coumaric acid [5].

In the case of flavonoids, thirteen compounds were detected and characterized, which were mainly kaempferol or quercetin derivatives. Thus, peak 6 was identified as quercetin diglucoside [22]. Moreover, it was possible to verify that peak 17 was quercetin-3-glucoside by comparison with the MS spectrum and retention time of the available commercial standard. With the same procedure, quercetin was associated to peak 30. Furthermore, three compounds (peaks 19, 21, and 24) gave the same deprotonated molecular formula C_23_H_21_O_13_ and were found in a range of time between 8.7 to 9.7 min, indicating that they are quercetin-3-*O*-(6-acetylglucoside) isomers, as previously reported [22]. Conversely, five kaempferol derivatives were also detected; all of them were bonded to one or more sugar moieties. Thereby, peak 12 showed a retention time at 7.3 min and an *m/z* at 755.2031. This information allowed its tentative identification as kaempferol rutinoside hexoside. Another kaempferol derivative was associated to peak 18, specifically kaempferol-3-rutinoside. It was only found in *Valenciana Temprana* MAE. In addition, peak 20 gave a retention time at 9.0 min and a molecular formula C_21_H_20_O_11_. This compound was associated to kaempferol-3-glucoside and it was verified with the available standard. Finally, two compounds (23 and 25) were found in *Italia*, *Kokuso*, and *Filipina* MAE and they were characterized as kaempferol-3-o-6-acetylglucopyranoside isomers according to the information available in literature [5,22]. To conclude with this phenolic group, two rutin isomers were related to peak 14 and 15. However, only the first isomer was only detected in *Kokuso* and *Filipina* MAE.

A total of thirteen fatty acid derivatives were found, many of them were isomers of hydroxylated fatty acids. For instance, peaks 34, 35, and 36 gave the same molecular formula and they were eluted at similar retention time, so they were identified as linoleic acid hydroperoxide isomers. Moreover, two isomers of hydroxy-octadecatrienoic acid were also detected (peak 46 and 47). All of these compounds were previously found in *M. nigra* [24]. Additionally, a derivate form of hydroxy-octadecatrienoic acid was found at 20.6 min. The compound 48 presented a molecular formula of C_18_H_28_O_3_ and was tentatively identified as hydroxyperoxy-octadecatrienoic acid. Besides, compound 50 gave a similar formula (C_18_H_31_O_3_), which, together with data suitable in literature, was possible to characterize as hydroxy- octadecadienoic acid [24]. Dihydroxy-eicosatrienoic acid, another unsaturated fatty acid derivative, was characterized to peak 44 that was only found in *Filipina* MAE. Its assignation was based on the presence of fatty acids in plants extracts of the same genus [24]. Peak 55 and 56 were found in all MAEs. They were characterized as linolenic acid and linoleic acid, respectively, since both of them have previously been described in *M. nigra* [24]. Furthermore, three saturated fatty acids were also found, one of them was dioxo-penta-oxatritricontanedioic acid, which showed a molecular formula, C_28_H_50_O_11_. Peak **33**, the first eluted fatty acid, was attributed to undecenoic acid [24]. Finally, myristic acid was detected and associated to compound 51 since it showed a molecular formula, C_14_H_22_O_2_. These compounds were found in all studied extracts.

On the other hand, compounds 1, 13, 16, 42, and 54 could not be possible to classify in chemicals groups mentioned before. As Table 2 shows, their occurrence is not uniform. Thus, peaks 1 and 13, sugar and fumaroprotocetraric acid isomer 1, respectively, were present in all MAEs, whereas fumaroprotocetraric acid isomer 2 (peak 16) was found in *Italia* and *Filipina* MAE. Moreover, aceroside VIII (peak 42) was tentatively identified for the first time in *M. alba*, which was found in plants from the same class [25]. Finally, peak 54 which showed a *m*/*z* at 555.2819 was only found in the *Valenciana Temprana* genotype and was also detected in botanicals from the same order [26].

### 3.2. Quantitation of Polar Compounds in MAEs

With the purpose of determining the amount of phenolic and other polar compounds presented in the MAEs nine commercial standards (quinic acid, protocatechuic acid, 3,5, methoxy-4-hydroxy cinnamic acid, chlorogenic acid, rutin, kaempferol-3-rutinoside, quercetin-3-glucoside, kaempferol-3-glucoside, and quercetin) were used to elaborate the calibration curves. Thus, calibration curves were drawn using eight points at different concentrations resulting in regression coefficients higher than 0.991. Compound concentrations were calculated by interpolation, the peak area of compound in the pertinent calibration curve. Quinic acid, chlorogenic acid, rutin, quercetin-3-glucoside, kaempferol-3-rutinoside, kaempferol-3-glucoside, and quercetin were quantified by comparison with the response of their respective standards.

The other compounds were tentatively calculated according to similar structures. In this sense, protocatechuic acid hexoside was quantified with protocatechuic acid calibration curve. In addition, chlorogenic acid curves were used to determine the amounts of rosmarinic acid and caffeoylquinic acid hexoside, whereas dimethoxy-cinnamic acid and p-coumaric acid were quantified with 3, 5, methoxy-4-hydroxy cinnamic acid curve. The amounts of the rest of the flavonoids in the extract were calculated using quercetin-3-glucoside, kaempferol-3-rutinoside, or kaempferol-3-glucoside. It should be mentioned that the quantitation of the analytes in *M. alba* could differ considering the response of the standards.

Therefore, the quantitation was only an estimation of their real concentration. Thus, Table 3 summarizes the quantitative results obtained by HPLC-ESI-TOF-MS expressed in µg of compound/g of *M. alba* leaves and µg of compound/g of extract. In this sense, the total content for all phenolics was calculated as the sum of the individual compound concentrations. It is necessary to remark that the analyte concentrations expressed by µg of analyte/g of extract gave practically the same distribution and ANOVA tests results than those expressed in µg of compound/g of *M. alba* leaves. Additionally, an analysis of variance (ANOVA) was made in order to know the significant differences among MAE.

Concerning the total phenolic content, it ranged from 58474 ± 2021 to 19171 ± 877 µg of analyte/g of *M. alba* leaves, where *Kokuso* was the MAE with highest amounts, whereas *Valenciana Temprana* had the lowest value. In this scenario, phenolic acids were the most abundant compounds in all extracts. Overall, chlorogenic acid isomers, specifically cryptochlorogenic acid, were the most representative compounds in all genotypes, with *Kokuso* as the one which reported the highest amounts of these compounds. These results are according with other previous studies [5].

On the other hand, protocatechuic acid glucoside was found in higher amounts in *Italia* MAE (4241 µg of analyte/g of *M. alba* leaves). The ANOVA test related to this compound revealed significant differences with regard to the rest of genotypes. Moreover, rosmarinic acid concentration displayed significant higher values in *Filipina* (2981 µg of analyte/g of *M. alba* leaves) compared with the rest of genotypes. Considering the rest of phenolic acids, dimethoxy cinnamic acid showed higher amounts in *Valenciana temprana* and *Filipina* (1077 and 1018 µg of analyte/g of *M. alba* leaves, respectively) genotypes than the others. On the other hand, *p*-coumaric acid revealed lower amounts in all genotypes, excluding *Valenciana temprana* which was the only variety that contained caffeoylquinic acid hexoside.

In spite of flavonoids being the most varied chemical group, their quantities were lower than the rest of the groups due to some compounds that were not found in all genotypes. For instance, quercetin dihexoside was only found in *Filipina* (94.9 µg of analyte/g of *M. alba* leaves), whereas quercetin acetyl glucoside isomers were not found in *Valenciana Temprana*. In this sense, quercetin acetyl glucoside isomer 1 was the major flavonoid found, showing significant differences in *Kokuso* (3353 µg of analyte/g of *M. alba* leaves) compared to *Filipina* and *Italia* (2872 and 694 µg of analyte/g of *M. alba* leaves, respectively). Concerning quercetin-3-glucoside, it was detected in higher amounts in *Filipina* (3092 µg of analyte/g of *M. alba* leaves). Moreover, rutin isomer 2 was the major flavonoid quantified in *Valenciana Temprana* and *Kokuso* (2977 and 2625 µg of analyte/g of *M. alba* leaves, respectively). These flavonoids were determined in great amounts in other studies [5,27]. Lastly, the most concentrated kaempferol derivative was kaempferol acetylglucoside isomer 1, which ranged from 1367 to 605 µg of analyte/g of *M. alba* leaves.

Regarding quinic acid, not phenolic molecule, significant differences were found in *Filipina* and *Kokuso* genotypes with regard to the others, reaching a concentration up to 10,994 µg of analyte/g of *M. alba* leaves.

### 3.3. Radical Scavenging Activity of the M. alba Extracts

Following the characterization of the MAE, the study of the in vitro antioxidant activity was carried out by measuring the total radical scavenging activity against DPPH, a widely used assay for the evaluation of antioxidant activity of biological samples. The results revealed that *Filipina* MAE showed the highest antioxidant capacity (441 ± 46 µmol AAE/g MAE), followed by *Kokuso* 431 ± 30 µmol AAE/g MAE) and *Italia* MAE (424 ± 39 µmol AAE/g MAE). Finally, a significantly lower scavenging activity was observed in *Valenciana Temprana* extract with 290 ± 21 µmol AAE/g MAE (ANOVA test, *p* < 0.05). The observed in vitro radical scavenging activity of the four MAEs correlates well with the total phenolic content of the different extracts as presented in Table 3.

### 3.4. Antioxidant Activity of the M. alba Extracts on C. elegans

**Figure 2:**
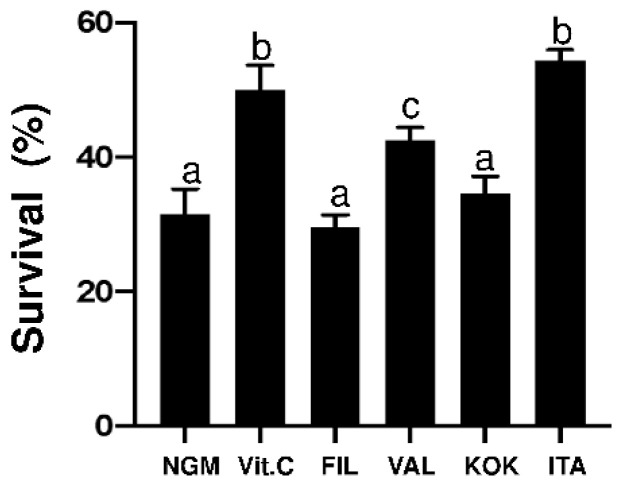
*C. elegans* survival (%) in the different conditions assayed (100 µg/mL) of the *Morus alba* leaves extracts( MAE) of different genotypes.

In a previous screening performed by our research group, a range of concentrations from 1 to 400 µg/mL of the different MAEs were evaluated, with the concentration of 100 µg/mL being selected as the optimal dose (data not shown). The effects of the MAE genotypes upon oxidative stress was determined in *C. elegans* by measuring worm viability after a 5-h-long exposure to 3 mM H_2_O_2_. The MAE-induced protective effects were compared to the effect exerted by the control *E. coli* OP50. The results showed a significant higher protective effect of *Italia* MAE similar to vitamin C positive control of worm survival (*p* < 0.05) and 23% higher than worm survival in the NGM control (*p* < 0.05) (Figure 2). *Valenciana Temprana* genotype showed a significant increase of protective effect (11% higher than worm survival in control condition), whereas *Kokuso* and *Filipina* genotypes did not show protective effect when compared to the control condition (Figure 2).

### 3.5. Effects of MAEs on Body Weight, Glucose Tolerance Test, Fat Accumulation, and Plasma Biochemical Profile

**Figure 3:**
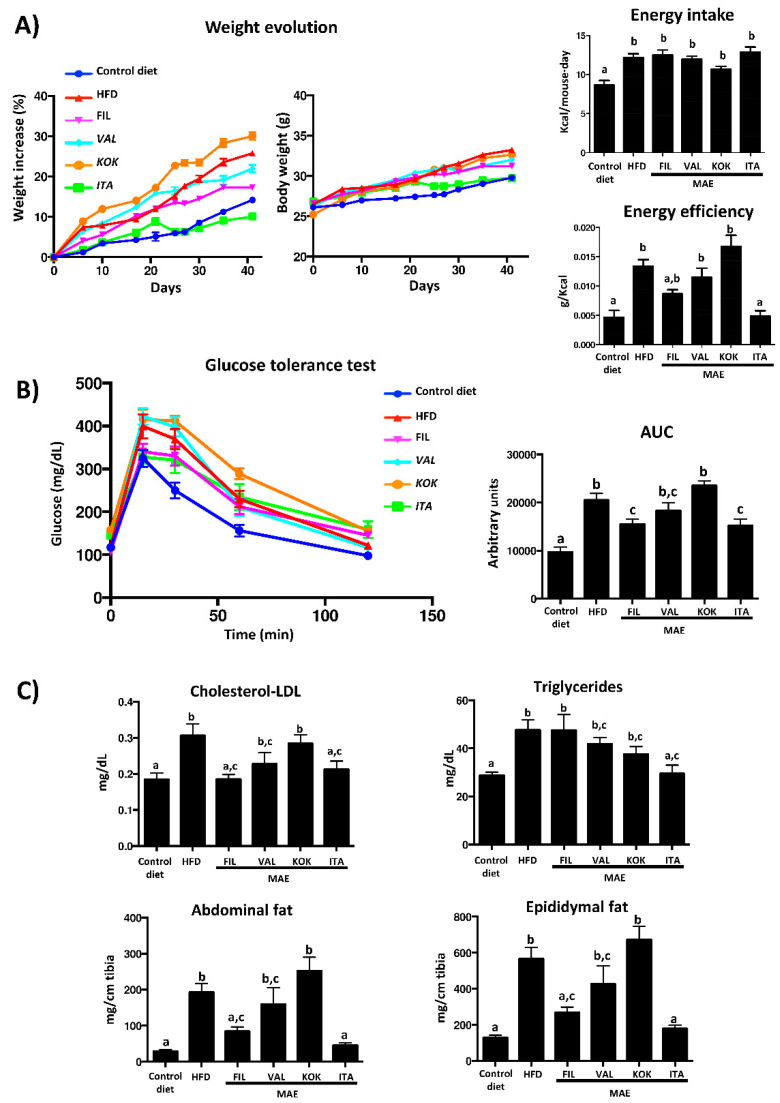
Effects of different *Morus alba* genotypes (*Filipina*, *Valenciana temprana*, *Kokuso*, and *Italia*) administration on (**A**) morphological changes (body weight evolution, energy efficiency, and energy intake); (**B**) glucose tolerance test and area under the curve (AUC); and (**C**) cholesterol-LDL plasma levels, triglycerides, and epididymal and abdominal fat. Data are expressed as means ± SEM (*n* = 10). Groups with different letters statistically differ (*p* < 0.05).

HFD consumption resulted in an increased body weight gain in the control obese group over the 42-day period when compared to the control group that received a standard diet. When HFD-fed mice were administered *Kokuso* or *Valenciana Temprana* MAE, no significant modifications were obtained in weight gain evolution in comparison with the corresponding control group, whereas *Filipina* and *Italia* produced a less pronounced weight gain, which was significantly different from control obese mice from days 37 and 25 onwards, respectively (Figure 3A).

Of note, no significant differences in energy intake were observed among groups receiving HFD (Figure 3A); however, only the treatment with *Italia* MAE significantly reduced the energy efficiency in comparison with untreated HFD-fed mice, obtaining similar values to those found in non-obese mice (Figure 3A).

These results would indicate that this extract did not reduce food intake but prevented the deleterious impact that HFD intake had on weight gain evolution in obese mice, as well as the obesity-associated metabolic disorders. Thus, the glucose tolerance test was performed one week before the sacrifice of mice, and the results revealed that plasma glucose levels reached a peak at 15 min after the administration of glucose in all experimental groups, and these values progressively decreased to the pre-prandial levels at the end of the assay.

As expected, the glycemia was higher in HFD-fed mice than in control diet-fed group; however, when HFD-fed mice were treated with *Filipina* or *Italia* MAE, the plasma glucose levels were lower in comparison with obese control mice and the normal levels were obtained earlier, thus reducing significantly the area under the curve (AUC) (Figure 3B). The beneficial effects on glucose metabolism exerted by these two genotypes were also associated with an improvement in lipid profile, since they were able to significantly reduce cholesterol-LDL levels in comparison with control HFD-fed mice; however, only *Italia* MAE significantly ameliorated the plasma triglycerides content (Figure 3C).

**Figure 4:**
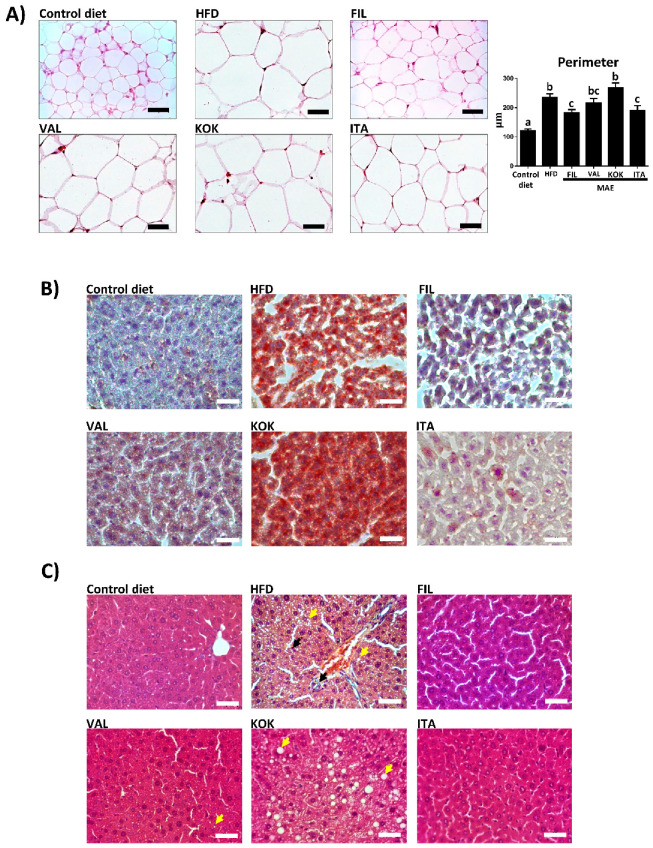
Effects of *Morus alba* genotypes (*Filipina*, *Valenciana temprana*, *Kokuso*, and *Italia*) administration on: (**A**) epididymal adipose tissue, analyzed by hematoxylin and eosin staining (scale bars = 40 μm) and on perimeter of epididymal adipocyte (Data are expressed as means ± SEM (*n* = 10) and groups with different letters statistically differ (*p* < 0.05)); (**B**) liver tissue stained with oil red and hematoxylin; and (**C**) liver tissue sections stained with hematoxylin and eosin (yellow arrows indicate the presence of lipid vacuoles in the cytoplasm of hepatocytes and black arrows indicate infiltrations of immune cells) in high fat diet (HFD)-fed mice.

Moreover, the increase of the abdominal and epididymal fat deposits observed in control obese mice was significantly lessened in obese mice treated with *Filipina* or *Italia* MAE, which correlated with the beneficial impact of these extracts on body weight evolution (Figure 3C). In fact, when the histological sections from epididymal fat tissue were evaluated, this tissue showed hypertrophy in untreated HFD-fed mice when compared with control diet-fed mice. However, the treatment with *Filipina* or *Italia* MAE reduced the size of the adipocytes when compared with control obese mice (Figure 4A).

Of note, the altered lipid metabolism was corroborated by the fat accumulation in liver, as evidenced in the histological analysis. When the liver sections were stained with oil red, which marks the lipids droplet accumulation, the red coloration was greater in samples from control obese mice than in those from non-obese mice (Figure 4B). Similarly, the increased fat deposition in liver was also observed when these sections were stained with hematoxylin and eosin, since the number of fat drops observed was higher in the cytoplasm of hepatocytes form HFD-fed mice (Figure 4C). Again, the administration of *Filipina* or *Italia* MAE to obese mice ameliorated the development of hepatic steatosis that occurred in the rest of groups of mice fed with HFD (Figure 4B,C).

### 3.6. Effects of MAEs on Systemic Inflammatory Response

**Figure 5:**
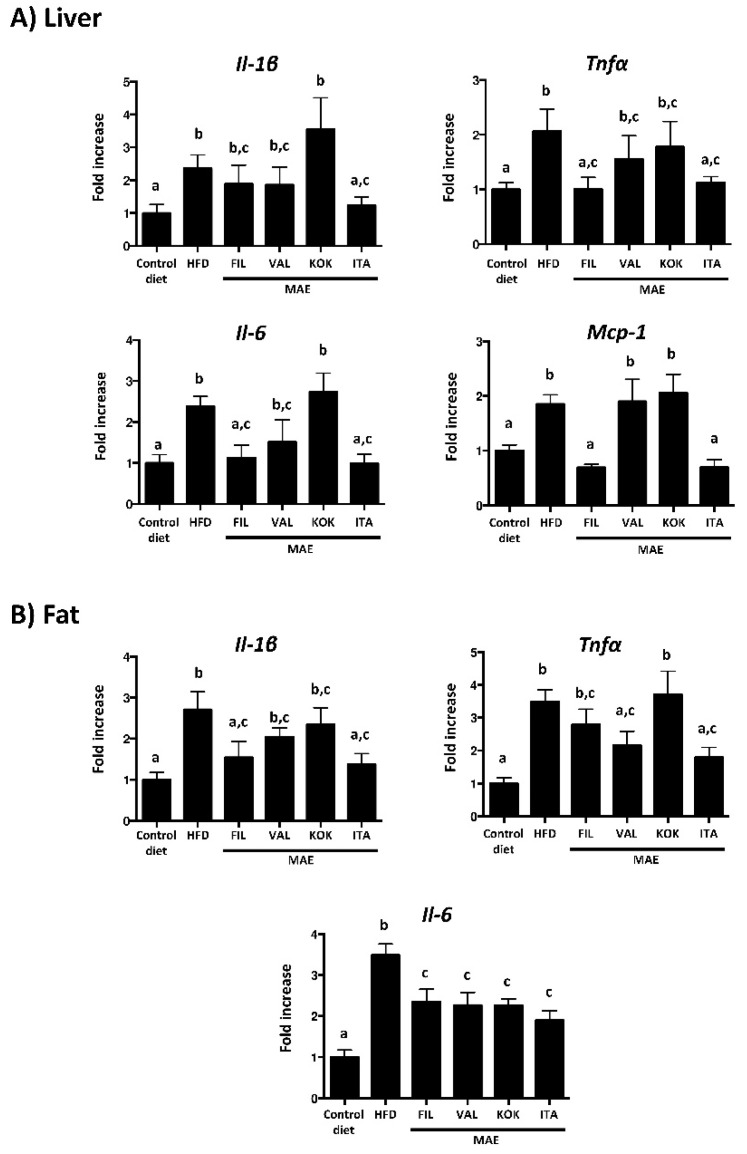
Effects after administration of *Morus alba* extracts *(*genotypes *Filipina*, *Valenciana temprana*, *Kokuso*, and *Italia*) in high fat diet (HFD)-fed mice: (**A**) on gene expression of *Il-1ß*, *Tnf-α*, *Il-6*, and *Mcp-1* in liver; (**B**) on gene expression of *Il-1ß*, *Tnf-α*, and *Il-6* in fat, analyzed by real time qPCR. Data are expressed as means ± SEM (*n* = 10). Groups with different letters statistically differ (*p* < 0.05).

Accumulating evidence indicates that obesity causes chronic low-grade inflammation, which clearly contributes to systemic metabolic dysfunctions that characterize metabolic syndrome [28,29]. This inflammatory status has been also observed in the present study, since mice fed HFD showed increased mRNA levels of the proinflammatory cytokines in hepatic and adipose tissues, including *Tnf-α*, *Il-1β*, *Il-6*, and the chemokine monocyte chemotactic protein-1 (*Mcp-1*). The ability of MAE to reduce weight gain, thus reducing obesity in these HFD-fed mice, can be the main contribution to ameliorate the systemic inflammatory response observed in the present study, especially when the *Italia* genotype is considered. This beneficial impact on obesity-associated inflammation can explain the improvement of glucose and lipid metabolism, since these pro-inflammatory cytokines have been correlated to an increment of lipolysis and fatty acid oxidation, thus amplifying the inflammatory process and facilitating insulin resistance (Figure 5) [30].

**Figure 6:**
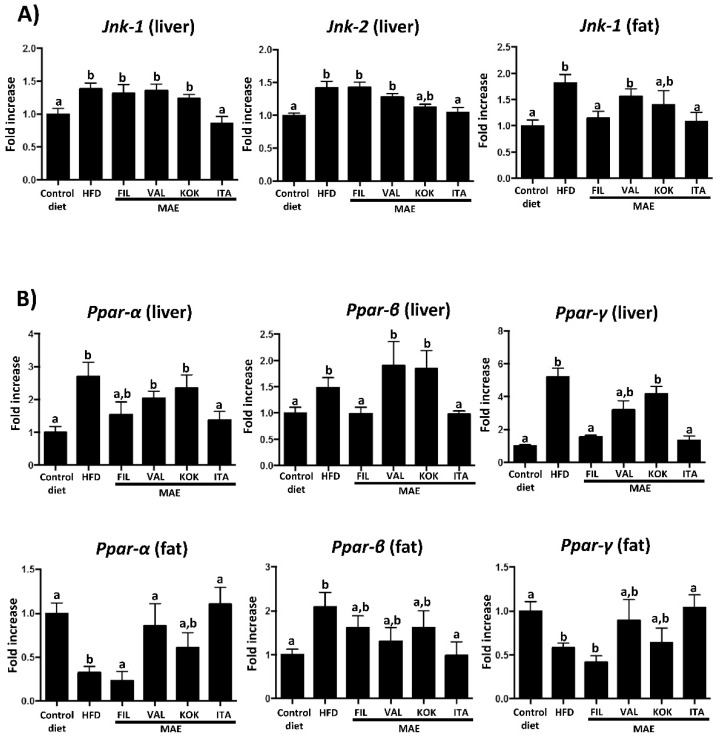
Effects after administration of *Morus alba* extracts *(*genotypes *Filipina*, *Valenciana temprana*, *Kokuso*, and *Italia*) in high fat diet (HFD)-fed mice: (**A**) gene expression of *Jnk-1* and *Jnk-2*; (**B**) gene expression of *Ppar-α*, *Ppar-β*, and *Ppar-γ*, analyzed by real time qPCR. Data are expressed as means ± SEM (*n* = 10). Groups with different letters statistically differ (*p* < 0.05).

C-Jun NH2-terminal kinases (JNKs) are members of the mitogen-activated protein kinase (MAPK), and it has been reported the important role that JNKs play in the development of obesity-induced inflammation, impaired glucose tolerance, and hepatic steatosis [31]. In fact, the intake of HFD increased the expression of *Jnk-1* and *Jnk-2* in liver and *Jnk-1* in fat; of note, only the administration of *Italia* MAE significantly reduced their expression (Figure 6A).

Peroxisome proliferator-activated receptors (PPARs) are ligand-activated transcription factors involved in the regulation of different biological processes such as lipid and glucose metabolism, as well as inflammation [32]. Diet-induced obesity produced an alteration in the expression of *Ppar α*, *β* and *γ* in liver and fat, and the treatment with *Italia* MAE normalized their mRNA levels (Figure 6B). PPAR-γ has an important role in the control of adipogenesis and lipid metabolism, inflammation, and insulin function, and its altered expression has been previously associated with obesity and diabetes [33]. Thus, one of the beneficial effects of the MAE would be related to the reduction of *Ppar-γ* levels in the liver as a consequence of the lower steatosis due to the minor weight gain. Similarly, the upregulation of *Ppar-α* in fat produced by this extract could be responsible for the improvement of insulin sensitivity and the increase of the glucose uptake in this tissue [34]. Of note, different types of adipose tissue have been reported, including white adipose tissue (WAT) and brown adipose tissue (BAT).

The increased accumulation of WAT in the body is associated with obesity-related inflammatory response; on the contrary, the formation of BAT, due to its thermogenic capacity, has been reported to ameliorate obesity [35]. More recently, a third type of adipose tissue has been also identified, beige adipose tissue, which can be derived from WAT, and, similarly to BAT, it is able to produce heat from glucose and fat due to the higher expressions of thermogenesis and lipolysis genes [36]. PPAR-γ is a transcriptional regulator of fat differentiation that has been proposed to participate in the browning of WAT [37]. In the present study, the administration of *Italia* MAE significantly counteracted the reduced expression of *Ppar-γ*, associated with obesity, which could be indicative of the browning of WAT in treated obese mice (Figure 6B), and the subsequent improvement of the metabolic response of adipose tissue in these mice.

Leptin and adiponectin are two adipokines involved in the balance regulation between energy intake and expenditure, and their expression, as well as the associated signaling in target organs, have been reported to be altered in obesity [38]. Leptin is considered as a proinflammatory adipokine, being involved in the activation of immune cells in hepatic and adipose tissues, and promotion of insulin resistance [39], and a situation of leptin resistance is a common feature in obesity, which is characterized by increased levels of Leptin, together with reduced expression of its receptor [40,41].

**Figure 7:**
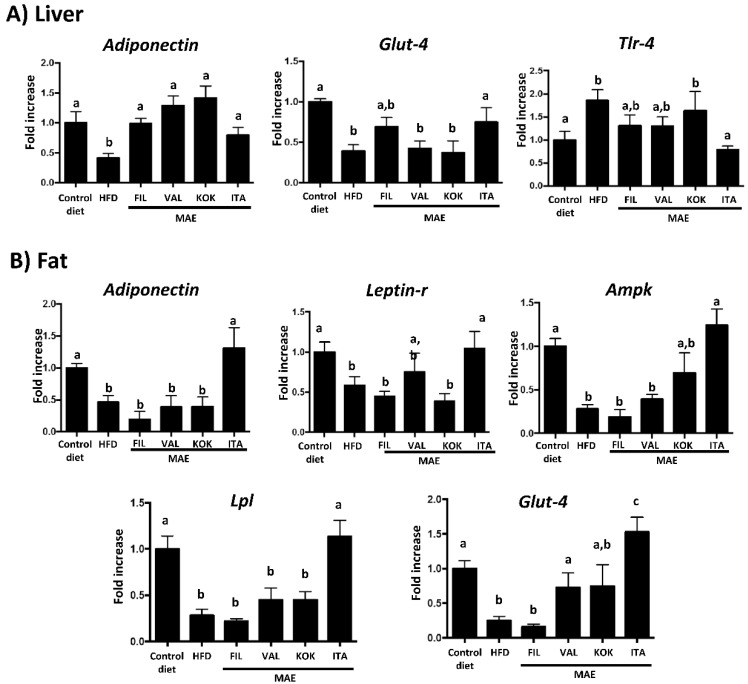
Effects after administration of *Morus alba* extracts *(*genotypes *Filipina*, *Valenciana temprana*, *Kokuso*, and *Italia*) in high fat diet (HFD)-fed mice: (**A**) gene expression of *Adinopectin*, *Glut-4*, and *Tlr-4* in liver; (**B**) gene expression of *Adinopectin*, *Leptin-r*, *Ampk*, *Lpl*, and *Glut-4* in fat, analyzed by real time qPCR. Data are expressed as means ± SEM (*n* = 10). Groups with different letters statistically differ (*p* < 0.05).

This has been evidenced in the present study, since control HFD-mice showed a decreased expression of leptin receptor in fat tissue, and, significantly, *Italia* MAE administration to HFD-fed mice improved its expression in this tissue (Figure 7). Unlike leptin, adiponectin is usually associated with anti-inflammatory actions in obesity [42]. In fact, the expression of adiponectin was significantly reduced in both liver and fat tissue from HFD-fed mice (Figure 7), closely related with the obesity-associated inflammatory status in obese mice. In comparison with control obese mice, the administration of all MAEs increased the mRNA levels of this adipokine in liver, whereas only *Italia* significantly increased the expression of adiponectin in fat tissue (Figure 7). Moreover, adiponectin exerts its action through AMP-activated protein kinase (AMPK), which has a relevant role in the control of inflammation and energy expenditure in obese individuals. This enzymatic complex stimulates catabolic pathways and blocks anabolic pathways, thus increasing energy expenditure [43]. Consequently, control HFD fed mice showed a reduced *Ampk* gene expression when compared to those mice fed with a standard diet; only the treatment with *Italia* MAE increased the mRNA levels of this kinase (Figure 7B), an effect that could also contribute to the beneficial actions of this MAE on inflammatory status and on energy efficiency observed in obese mice.

Lipoprotein lipase (LPL) is an enzyme that hydrolyses triglycerides, and its expression and/or activity has been reported to be reduced in obesity, thus leading to hypertriglyceridemia [44,45]. In fact, the present study revealed that the expression of Lpl was significantly reduced in the adipose tissue from control mice fed HFD, in comparison with non-obese mice. Of note, the administration of *Italia* MAE was able to restore Lpl mRNA levels to values similar to non-obese mice (Figure 7B), and this could explain that this MAE was the only one that significantly reduced plasma triglycerides in HFD-fed mice.

In addition, it is well known that glucose uptake in different tissues requires the presence of the membrane transporters, like GLUT-4, of which expression and function can be impaired in metabolic conditions like insulin resistance and diabetes [46]. In fact, in the present study, Glut-4 gene expression is reduced in fat tissue and liver in obese mice, similarly to that previously reported in similar experimental models of obesity [47], thus decreasing the glucose uptake and resulting in hyperglycemia.

Except *Filipina* MAE, all the extracts have shown a beneficial impact on Glut-4 expression in fat tissue; however, only *Italia* MAE also significantly upregulated the expression of Glut-4 gene in liver (Figure 7). Once again, the better profile of this MAE could contribute to its highest efficacy to ameliorate the glycemic and lipid metabolic alterations observed, as well as to downregulate the associated systemic inflammatory response in obese mice. Closely related to this, it is interesting to note that this situation has been associated to an increase in bacterial lipopolysaccharide in plasma that causes metabolic endotoxemia [48].

In fact, some studies have demonstrated the correlation between increased LPS plasma levels and dysregulation of toll-like receptor 4 (TLR-4) signaling, specifically with an increase in Tlr-4 gene expression [49]. This receptor recognizes LPS and triggers mechanisms that increase the expression of pro-inflammatory cytokines [50]. As expected, the mRNA levels of Tlr-4 in liver significantly increased in mice fed an HFD in comparison with the control lean mice, which were significantly reduced in obese mice administered with *Italia* MAE (Figure 7A), thus ameliorating the endotoxemia-associated inflammatory status that takes place in obesity.

**Figure 8:**
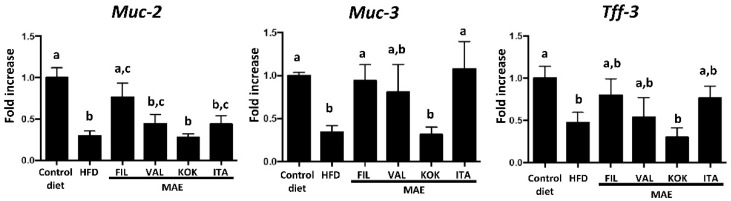
Effects after administration of *Morus alba* extracts *(*genotypes *Filipina*, *Valenciana temprana*, *Kokuso*, and *Italia*) in high fat diet (HFD)-fed mice on gene expression of intestinal barrier integrity markers, *Muc-2*, *Muc-3*, and *Tff-3*, analyzed by real time qPCR. Data are expressed as means ± SEM (*n* = 10). Groups with different letters statistically differ (*p* < 0.05).

Finally, this obesity-associated metabolic endotoxemia may have its origin in an increase in intestinal permeability that takes place in obesity, which would facilitate the access of microbial components from intestinal lumen to systemic circulation [51,52]. The altered epithelial barrier function can be observed in this study since the expression of proteins involved in intestinal integrity was reduced in obese mice in comparison with mice fed a standard diet (Figure 8).

When the impact of the different MAEs was evaluated, *Filipina* showed the best profile in restoring the colonic expression of the markers related with the intestinal barrier function, as it significantly increases the expression of genes such as *Muc-2*, *Muc-3*, and *Tff-3* (Figure 8). This improvement was also observed in obese mice receiving *Italia* MAE, although the significant differences in comparison with control HFD-fed mice were only obtained with the expression of *Muc-3*, whereas only a trend was obtained with the other marker of intestinal integrity. These effects on epithelial barrier function could be associated with the prevention of metabolic endotoxemia that takes place in obesity, thus reducing the inflammatory status and contributing to the beneficial effects observed in this experimental model of metabolic syndrome.

Considering all the results, the greater efficacy shown by *Filipina* and, especially, *Italia* MAE among the different genotypes evaluated must be due to the specific chemical composition of each extract. In fact, it has been previously proposed that phenolic compounds are able to enhance thermogenesis, thus regulating the altered energy expenditure observed in obesity; in consequence, further studies are required to evaluate the impact of these extracts on brown and white fat tissue activities [53,54]. Additionally, the presence of protocatechuic acid is significantly higher in *Italia* MAE. This compound has been reported to show anti-obesity, antidiabetic, antiatherosclerotic, and anti-inflammatory properties, being effects associated with its ability to increase the expression and membrane translocation of the glucose transporter *Glut-4*, thus facilitating adipocyte glucose uptake; moreover, protocatechuic acid was able to upregulate the levels of adiponectin, *Ppar-γ* and *Ampk*, which can also account for the thus ameliorating of the insulin resistance and obesity-associated disorders [55,56]. When *Filipina* MAE is considered, quercetin-3-glucoside is one of the main analytes detected, with reported beneficial effects against experimental obesity, given its ability to improve the altered lipid profile and to ameliorate the higher weight gain in obese mice, as well as to modulate the expression of genes involved in lipid metabolism and adipocyte differentiation [57,58]. Moreover, fumaroprotocetraric acid isomer 2 was found in the two most active MAEs, *Filipina* and *Italia*; this fatty acid possesses antioxidant and antimicrobial properties, which may be also involved in their beneficial effects observed [59].

## 4. Conclusions

In conclusion, *Morus alba* leaf extracts from different genotypes show a positive impact in HFD-mice by reducing the body weight gain, in which the antioxidant or anti-inflammatory properties exerted by these extracts are involved. However, these beneficial effects depend on the genotype. Although the *Italia* genotype does not present an outstanding total polyphenol content and in vitro radical scavenging activity, it showed the greatest protective effect against the acute oxidative stress in the *C. elegans* model, and the highest anti-inflammatory efficacy in HFD-induced obesity, followed by the *Filipina* genotype. These extracts could be used as a complementary treatment for obesity and metabolic syndrome, mainly due to the presence of particular bioactive compounds in their extracts, including protocatechuic acid and quercetin-3-glucoside, with antioxidant and anti-inflammatory properties.

## Figures and Tables

**Table 1:** Primer sequences used in real-time PCR assays.

Gen	Sequence 5′-3′	Sequence Accession Number	Annealing Temperature (°C)
*Gapdh*	D: CCATCACCATCTTCCAGGAG R: CCTGCTTCACCACCTTCTTG	AK002273.1	60
*Il-1β*	D: TGATGAGAATGACCTCTTCT R: CTTCTTCAAAGATGAAGGAAA	AK225002.1	60
*Tnf-α*	D: AACTAGTGGTGCCAGCCGAT R: CTTCACAGAGCAATGACTCC	AK159989.1	60
*Il-6*	D: TAGTCCTTCCTACCCCAATTTCC R: TTGGTCCTTAGCCACTCCTTCC	AK152189.1	60
*Jnk-1*	D: GATTTTGGACTGGCGAGGACT R: TAGCCCATGCCGAGAATGA	BC053027.1	60
*Jnk-2*	D: TTGTGCTGCTTTTGATACAGTTCTTGGG R: CTGGAAAGAGCTCTTCAAATTTGAT	AK031959.1	62
*Mcp-1*	D: AGCCAACTCTCACTGAAG R: TCTCCAGCCTACTCATTG	AK150937.1	55
*Leptin*	D: TTCACACACGCAGTCGGTAT R: GCTGGTGAGGACCTGTTGAT	KX255818.1	60
*Leptin-r*	D: GCAGTCCTCAGTGGCACCTC R: CACCGTGGGGCTACTGGAGAG	AK143989.1	60
*Ampk*	D: GACTTCCTTCACAGCCTCATC R:CGCGCGACTATCAAAGACATACG	AK160612.1	60
*Ppar-α*	D: AGGCTGTAAGGGCTTCTTTCG R: GGCATTTGTTCCGGTTCTTC	AK035676.1	62
*Ppar-β*	D: TAGGACTGGTGATCTGTGAG R: TACAAGTGAGTGGGAGAGAG	AK028464.1	60
*Ppar-γ*	D: CAGTACAGCCCCGATGACTC R: GAAAGCTCGTCCACGTCAG	AH013273.2	62
*Glut-4*	D: GAGAATACAGCTAGGACCAGTG R:TCTTATTGCAGCAGCGCCTGAG	AK137607.1	62
*Tlr-4*	D: GCCTTTCAGGGAATTAAGCTCC R: AGATCAACCGATGGACGTGTAA	JX878359.1	60
*Adiponectin*	D: GATGGCAGAGATGGCACTCC R: CTTGCCAGTGCTGCCGTCAT	AK003138.1	56
*Lpl*	D: TTCCAGCCAGGATGCAACA R: GGTCCACGTCTCCGAGTCC	AK150328.1	60
*Muc-1*	D: GCAGTCCTCAGTGGCACCTC R: CACCGTGGGCTACTGGAGAG	BG005441.1	60
*Muc-2*	D: GCAGTCCTCAGTGGCACCTC R: CACCGTGGGGCTACTGGAGAG	AK008250.1	60
*Muc-3*	D: CGTGGTCAACTGCGAGAATGG R: CGGCTCTATCTCTACGCTCTCC	AK136468.1	60
*Occludin*	D: ACGGACCCTGACCACTATGA R: TCAGCAGCAGCCATGTACTC	AK019880.1	56
*Tff-3*	D: CCTGGTTGCTGGGTCCTCTG R:GCCACGGTTGTTACACTGCTC	D38410.1	60
*Zo-1*	D: GGGGCCTACACTGATCAAGA R: TGGAGATGAGGCTTCTGCTT	AK144506.1	56

**Table 2:** Phytochemical composition of the analyzed *M. alba* leaves.

Cmpd	RT (min)	*m*/*z* Exp	Molecular Formula	Tentative Identification	Presence			
					I	K	F	V
**Organic Acids**								
2	1.9	191.0572	C_7_H_11_O_6_	Quinic acid	+	+	+	+
**Benzoic Acids**								
3	5.1	315.0722	C_13_H_15_O_9_	Protocatechuic acid hexoside	+	+	+	+
4	5.6	459.1122	C_19_H_23_O_13_	Parishin E			+	
26	10.5	469.1704	C_22_H_29_O_11_	Rengyoside D	+			
28	10.8	135.0455	C_8_H_7_O_2_	Methyl salicyl aldehyde				+
36	14.5	221.1179	C_13_H_17_O_3_	Hexyl salicylate	+	+	+	+
52	24.3	265.1448	C_15_H_21_O_4_	Gingerol	+	+	+	+
**Cinnamic Acids**								
5	5.8	353.0881	C_16_H_17_O_9_	Neochlorogenic acid	+	+	+	+
7	6.6	353.0865	C_16_H_17_O_9_	Chlorogenic acid	+	+	+	+
8	6.7	353.0876	C_16_H_17_O_9_	Cryptochlorogenic acid	+	+	+	+
9	6.9	359.0758	C_18_H_15_O_8_	Rosemarinic acid	+	+	+	+
11	7.2	353.085	C_16_H_17_O_9_	Chlorogenic acid isomer	+	+	+	+
22	9.3	515.1186	C_25_H_23_O_12_	Caffeoylquinic acid hexoside				+
31	12.1	207.0659	C_11_H_11_O_4_	Dimethoxy-cinnamic acid	+	+	+	+
32	12.6	163.0391	C_9_H_7_O_3_	Coumaric acid	+	+	+	
**Flavonoids**								
6	6.2	625.1420	C_27_H_29_O_17_	Quercetin dihexoside	+	+	+	+
12	7.3	755.2031	C_33_H_39_O_20_	Kaempferol rutinoside hexoside				+
14	7.7	609.1469	C_27_H_29_O_16_	Rutin isomer 1		+	+	
15	8.0	609.1467	C_27_H_29_O_16_	Rutin isomer 2	+	+	+	+
17	8.4	463.0879	C_21_H_19_O_12_	Quercetin-3-glucoside	+	+	+	+
18	8.6	593.1510	C_27_H_29_O_15_	Kaempferol-3-*o*-rutinoside	+	+	+	+
19	8.7	505.0990	C_23_H_21_O_13_	Quercetin-3-*O*-(6-acetylglucoside) isomer 1	+	+	+	
20	9.0	447.0908	C_21_H_19_O_11_	Kaempferol 3-*o-*glucoside	+	+	+	+
21	9.2	505.0997	C_23_H_21_O_13_	Quercetin-3-*O*-(6-acetylglucoside) isomer 2	+	+	+	
23	9.4	489.1040	C_23_H_21_O_12_	Kaempferol-3-*o*-6″-*o*-acetyl-β-d glucopyranoside isomer 1	+	+	+	
24	9.7	505.0997	C_23_H_21_O_13_	Quercetin-3-*O*-(6-acetylglucoside) isomer 3	+	+	+	
25	10.0	489.1040	C_23_H_21_O_12_	Kaempferol-3-*o*-6″-*o*-acetyl-β-d glucopyranoside isomer 2	+	+	+	
30	11.5	301.0357	C_15_H_9_O_7_	Quercetin	+	+	+	+
**Fatty Acids**								
33	13.1	183.1398	C_11_H_19_O_2_	Undecenoic acid	+	+	+	+
34	13.7	309.2057	C_18_H_29_O_4_	Linoleic acid hydroperoxide isomer 1	+	+	+	+
35	13.9	309.2057	C_18_H_29_O_4_	Linoleic acid hydroperoxide isomer 2	+	+	+	+
37	15.7	309.2074	C_18_H_29_O_4_	Linoleic acid hydroperoxide isomer 3	+	+	+	+
44	18.6	337.2356	C_20_H_33_O_4_	Dihydroxy eicosatrienoic acid			+	
46	19.2	293.2161	C_18_H_29_O_3_	Hydroxy octadecatrienoic acid isomer 1	+	+	+	
47	19.5	293.2161	C_18_H_29_O_3_	Hydroxy octadecatrienoic acid isomer 2	+	+	+	
48	20.6	291.1949	C_18_H_27_O_3_	Hydroxyperoxy octadecatrienoic acid	+	+	+	
49	20.9	561.3249	C_28_H_49_O_11_	Dioxo-penta-oxatritriacontanedioic acid				+
50	21.2	295.2252	C_18_H_31_O_3_	Hydroxy octadecadienoic acid	+	+	+	
51	21.5	221.1530	C_14_H_21_O_2_	Myristic acid	+	+	+	+
55	29.4	277.2155	C_18_H_29_O_2_	Linolenic acid	+	+	+	+
56	33.0	279.2315	C_18_H_31_O_2_	Linoleic acid	+	+	+	+
**Others**								
1	1.9	341.1100	C_12_H_21_O_11_	Sugar	+	+	+	+
13	7.6	481.1332	C_22_H_25_O_12_	Fumaroprotocetraric acid isomer 1	+	+	+	+
16	8.3	481.1338	C_22_H_25_O_12_	Fumaroprotocetraric acid isomer 2	+		+	
42	17.7	593.2674	C_30_H_41_O_12_	Aceroside VIII	+	+	+	
54	27.1	555.2819	C_28_H_43_O_11_	Picrasinoside F				+
**Unknowns**								
10	7.1	431.1905	C_20_H_31_O_10_	Unk 1				+
27	10.5	459.2232	C_22_H_35_O_10_	Unk 2				+
29	11.2	357.0613	C_18_H_13_O_8_	Unk 3				+
38	16.0	721.3611	C_34_H_57_O_16_	Unk 4	+	+	+	+
39	16.4	721.3620	C_34_H_57_O_16_	Unk 5	+	+	+	+
40	17.0	562.3133	C_26_H_36_ N_13_O_2_	Unk 6	+	+	+	+
41	17.4	552.2667	C_24_H_42_NO_13_	Unk 7		+		
43	18.5	559.3124	C_28_H_47_O_11_	Unk 8	+	+	+	+
45	18.9	559.3105	C_28_H_47_O_11_	Unk 9	+	+	+	+
53	26.5	481.2502	C_25_H_37_O_9_	Unk 10			+	

I: *Italia*; K: *Kokuso*; F: *Filipina*; and V: *Valenciana Temprana*. (+) Presence in the Methanolic extract.

**Table 3:** Quantification of compounds from different *Morus alba* genotypes. Values expressed as µg of analyte/g of *M. alba* leaves ± SD.

Compound	*Italia*	*Filipina*	*Kokuso*	*Valenciana Temprana*
Quinic acid	3711 ± 49 ^b^	9341 ± 259 ^a^	10,994 ± 236 ^a^	1469 ± 55 ^c^
Protocatechuic acid glucoside	4241 ± 380 ^a^	1809 ± 126 ^b^	1368 ± 138 ^b,c^	905 ± 27 ^c^
Neochlorogenic acid	4430 ± 154 ^c^	5753 ± 43 ^b^	10,091 ± 737 ^a^	2260 ± 72 ^d^
Chlorogenic acid	5807 ± 101 ^b^	9089 ± 774 ^a^	5394 ± 274 ^b^	2345 ± 131 ^c^
Cryptochlorogenic acid	10,537 ± 733 ^b^	12,478 ± 82 ^a^	16,514 ± 32 ^a^	4602 ± 289 ^c^
Rosmarinic acid	1611 ± 87 ^b^	2981 ± 253 ^a^	1625 ± 85 ^b^	1343 ± 71 ^b^
Chlorogenic acid isomer	1162 ± 27 ^a^	1280 ± 19 ^a^	1288 ± 120 ^a^	99 ± 11 ^b^
Caffeoylquinic acid hexoside	ND	ND	ND	396 ± 13 ^a^
Dimethoxy cinnamic acid	194 ± 8 ^c^	1018 ± 33 ^a^	639 ± 107 ^b^	1077 ± 55 ^a^
p-coumaric acid	85 ± 5 ^b^	221 ± 5 ^a^	95 ± 9 ^b^	ND
Quercetin dihexoside	NQ	94.9 ± 0.2 ^a^	NQ	NQ
Rutin isomer 1	ND	66 ± 3 ^a^	ND	23.1 ± 0.4 ^b^
Rutin isomer 2	2343 ± 54 ^c^	2039 ± 46 ^d^	2625 ± 15 ^b^	2977 ± 69 ^a^
Quercetin-3- glucoside	274 ± 55 ^d^	3092 ± 214 ^a^	1302 ± 70 ^b^	702 ± 51 ^c^
Kaempferol-3-rutinoside	574 ± 5 ^b^	166 ± 8 ^d^	275 ± 8 ^c^	973 ± 33 ^a^
Quercetin acetylglucoside isomer 1	604 ± 23 ^c^	2872 ± 221 ^b^	3353 ± 108 ^a^	ND
Kaempferol-3-glucoside	141 ± 6 ^c^	675 ± 10 ^a^	232.7 ± 0.8 ^b^	NQ
Quercetin acetylglucoside isomer 2	NQ	1122 ± 9 ^a^	877 ± 22 ^b^	ND
Kaempferol acetylglucoside isomer 1	605 ± 27 ^c^	1133 ± 24 ^b^	1367 ± 33 ^a^	ND
Quercetin acetylglucoside isomer 3	NQ	NQ	NQ	ND
Kaempferol acetylglucoside isomer 2	95 ± 5 ^c^	576 ± 11 ^a^	435 ± 26 ^b^	ND
Quercetin	NQ	NQ	NQ	NQ
Total phenolic content	36,414 ± 1719 ^b^	55,806 ± 2140 ^a^	58,474 ± 2021 ^a^	19,171 ± 877 ^c^

NQ: Compound detected but below LOQ; ND: Compound not detected. Concentrations with the same type (a–c) did not present significant differences.
